# Empagliflozin Plays Vasoprotective Role in Spontaneously Hypertensive Rats via Activation of the SIRT1/AMPK Pathway

**DOI:** 10.3390/cells14070507

**Published:** 2025-03-29

**Authors:** Monika Kloza, Anna Krzyżewska, Hanna Kozłowska, Sandra Budziak, Marta Baranowska-Kuczko

**Affiliations:** 1 Department of Experimental Physiology and Pathophysiology, Medical University of Białystok, 15-222 Białystok, Poland; anna.krzyzewska@umb.edu.pl (A.K.); hanna.kozlowska@umb.edu.pl (H.K.); 41638@student.umb.edu.pl (S.B.); 2 Department of Clinical Pharmacy, Medical University of Białystok, 15-222 Białystok, Poland

**Keywords:** empagliflozin, SGLT2 inhibitors, sirtuin 1, AMPK, spontaneously hypertensive rat, vasoprotection

## Abstract

Empagliflozin (EMPA), a sodium-glucose co-transporter 2 (SGLT2) inhibitor, prevents endothelial dysfunction, but its effects on vascular tone in hypertension remain unclear. This study investigated whether EMPA modulates vasomotor tone via sirtuin 1 (SIRT1) and AMP-activated protein kinase (AMPK) pathways in spontaneously hypertensive rats (SHR) and controls (Wistar Kyoto rats, WKY). Functional (wire myography, organ bath) and biochemical (Western blot) studies were conducted on the third-order of the superior mesenteric arteries (sMAs) and/or aortas. EMPA induced concentration-dependent relaxation of preconstricted sMAs in both groups. In SHR, EMPA enhanced acetylcholine (Ach)-induced relaxation in sMAs and aortas and reduced constriction induced by phenylephrine (Phe) and U46619 in sMAs. The SIRT1 inhibitor (EX527) abolished EMPA’s effects on Ach-mediated relaxation and U46619-induced vasoconstriction, while AMPK inhibition reduced Ach-mediated relaxation and Phe-induced vasoconstriction. SHR showed increased SGLT2 and SIRT1 expression and decreased pAMPK/AMPK levels in sMAs. In conclusion, EMPA might exert vasoprotective effects in hypertension by enhancing endothelium-dependent relaxation and reducing constriction via AMPK/SIRT1 pathways. These properties could improve vascular health in patients with hypertension and related conditions. Further studies are needed to explore new indications for SGLT2 inhibitors.

## 1. Introduction

Flozins are sodium-glucose cotransporter type 2 (SGLT2) inhibitors and are a new generation of drugs intended for the treatment of type 2 diabetes [1]. Multiple randomized controlled trials and real-world data studies have demonstrated significant benefits for cardiovascular health, including nephro- and cardioprotection, both in diabetic and non-diabetic patients independent of initial risk factors [2,3,4] Therefore, the European Society of Cardiology indicates flozins as one of the pillars of therapy for patients with heart failure independent of left ventricular ejection fraction and chronic kidney disease [1].

Among the available flozins, we distinguish dapagliflozin, canagliflozin, ertugliflozin, sotagliflozin, and empagliflozin (EMPA) [5]. All SGLT2 inhibitors offer some common benefits, but EMPA stands out due to its long half-life, highest selectivity for SGLT2, and the best-documented cardiovascular benefits. It also has slightly stronger nephroprotective effects than other SGLT2 inhibitors, the best hemodynamic profile, and a lower risk of side effects [2,3,6] and has been shown to exert beneficial effects in vasculoprotective effect in different phases of diabetes disease progression, including in co-morbid conditions in preclinical and clinical studies [7].

The pleiotropic pharmacological mechanisms by which SGLT2 inhibitors, including EMPA, exert their cardiovascular and renal benefits are not fully understood but go far beyond blood glucose reduction [8,9]. Relatively little is known about their effects on the vasculature [10] and their impact on macro- and microvascular endothelial functions [7]. Thus far, EMPA has been demonstrated to directly benefit vascular health by enhancing vascular endothelial function, which involves reducing oxidative stress, inflammation, and arterial stiffness [7,11,12]. Moreover, it regulates endothelial cell processes such as proliferation, migration, differentiation, survival, and senescence [7,13]. Additionally, EMPA has been shown to relax the resistance mesenteric [10,14,15] and coronary arteries of Sprague Dawley rats [16], cardiac septal resistance, and renal conduit arteries of Wistar rats [10], and the rabbit’s aortas [17].

Recently, in a comprehensive overview of the SGLT2 inhibitor interaction network and its implications for specific effects, sirtuin 1 (SIRT1)-SGLT2 was identified among the top five interactions that are associated with key terms and signaling pathways related to the SGLT2 inhibitors and among others with a nicotinamide adenine dinucleotide (NAD)^+^-dependent deacetylase and AMP-activated protein kinase (AMPK) pathways [18]. Co-immunoprecipitation showed that SIRT1 colocalizes with and coprecipitates in endothelial cells with endothelial nitric oxide synthase (eNOS), suggesting that SGLT2 inhibitors promote its upregulation and binding to eNOS [19,20]. AMPK activity in endothelial cells can be regulated by various stimuli, including low ATP levels, as occurs in stressed conditions, and by increasing intracellular calcium through endogenous and pharmacological activators [21]. It was found that EMPA, via the SIRT1 modulation, can alleviate the experimental model of myocardial injury [22] and cardiac microvascular injury in diabetes [23]. SIRT1 may be involved in the post-myocardial injury remodeling process, possibly protecting it from apoptosis but inducing hypertrophy [24]. Both SIRT1 and AMPK are key regulators of cellular homeostasis and share mechanisms with EMPA in maintaining vascular tone and preventing endothelial dysfunction [25,26]. Dysregulation of these pathways is implicated in the progression of cardiovascular diseases, including hypertension, which remains the most significant risk factor for premature death worldwide, and comorbidity with diabetes, heart, and kidney diseases [27]. However, the specific role of EMPA in preventing vascular changes induced by hypertension through the SIRT1/AMPK pathways remains unclear.

Considering the above, EMPA has been shown to benefit vascular health. However, its effects on vascular function in spontaneously hypertensive rats (SHR) have not yet been studied. The interplay between SIRT1 and AMPK may represent a key mechanism underlying the vasoprotective effects of EMPA with the hypothesis that these compounds could improve endothelium-dependent vasorelaxation and reduce vasoconstriction effects. Therefore, this study aims to investigate whether SIRT1 and AMPK are involved in these vasoprotective effects of EMPA in SHR. By elucidating these mechanisms, the study may contribute to a deeper understanding of the therapeutic potential of EMPA and its implications for managing cardiovascular complications associated with hypertension.

## 2. Materials and Methods

### 2.1. Animals

Male 10–12 week-old SHR and Wistar Kyoto rats (WKY, 10 rats per group) that weighed 280–310 g were purchased from the Center for Experimental Medicine of the Medical University of Bialystok. All surgical procedures and experimental protocols were carried out following the European Directive (2010/63/EU) and Polish legislation. Our research does not require approval from the Animal Research Ethics Committee. According to Article 3 of the Act of 15 January 2015, under Polish law, euthanizing an animal solely to use its organs or tissues is not considered an experimental procedure requiring Ethics Committee approval. Animal studies were conducted by the ARRIVE guidelines [28]. The study was performed in compliance with the principles of replacement, refinement, or reduction (the 3Rs). Animals were housed at constant humidity (60 ± 5%) and temperature (22 ± 1 °C) and were kept under a 12:12 h light–dark cycle. They were fed standard pelleted rat chow and tap water ad libitum.

### 2.2. Measurements of Blood Pressure

Systolic blood pressure (SBP) was measured in conscious animals by a non-invasive tail-cuff method with the Non-Invasive Blood Pressure Controller (NIBP). For indirect SBP readings, the rats were placed in acrylic restrainers in a chamber at 35 °C for 10 min. The tail cuff and sensor were connected to NIBP Controller (ADInstruments, Dunedin, New Zealand), with the max mmHg set to 280 and the pulse range to 240–600 beats per minute. The signals from the pulse and pressure sensors were recorded and analyzed using LabChart 8.1.27 Pro (ADInstruments, Dunedin, New Zealand) with sampling set to 1 k/s. Animals with SBP equal to or higher than 150 mmHg [29] were considered hypertensive and underwent an organ bath, myography procedure, and biochemical evaluations [30].

### 2.3. Vessel Preparation

Male SHR and WKY rats were anesthetized with pentobarbitone sodium (300 µM/kg i.p.). Two-millimeter segments of the third-order branches of the superior mesenteric artery (sMAs) and the thoracic aortas were isolated and mounted in a Mulvany-Halpern-type wire myograph (MultiWire Myograph System DMT 620 M, Danish Myo Technology, Aarhus, Denmark) and in 10 mL organ baths (PIM 100RE, BIO-SYS-TECH, Białystok, Poland), respectively, following previously described methods [30].

**Figure 1:**
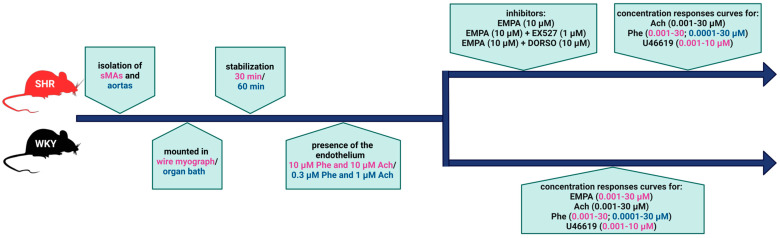
The scheme of the experimental protocol on third-order superior mesenteric arteries (sMAs) and thoracic aortas. Ach—acetylcholine; DORSO—dorsomorphin, inhibitor of AMP-activated protein kinase AMPK [26]; EMPA—empagliflozin; EX527—inhibitor of SIRT1 [31]; Phe—phenylephrine; U46619—synthetic thromboxane A2 (9,11-dideoxy-9α,11α-methanoepoxy prostaglandin F2α); WKY—Wistar Kyoto rats; SHR—spontaneously hypertensive rats. Created with BioRender.com.

After an equilibration period, endothelial integrity was assessed by pre-contracting the arterial rings with the α1-adrenoceptor agonist phenylephrine (Phe; sMAs: 10 μM and aortas: 0.3 μM), followed by relaxation with acetylcholine (Ach; 10 or 1 μM, respectively). A relaxation response of at least 90% to Ach was considered indicative of an endothelium-intact vessel. After washout, the cumulative concentration-response curves (CRCs) were constructed by the cumulative addition of appropriate agonists with or without specific inhibitors (with only one protocol performed on each arterial ring). The experimental design is presented in Figure 1.

### 2.4. Western Blot

The method was performed as described previously in Krzyżewska et al. [32]. Briefly: sMAs and aortas were homogenized in a solution containing mammalian protein extraction reagent (MPER, Thermo Scientific, Waltham, MA, USA) and the cocktail of phosphatase and protease inhibitors (Roche Diagnostics GmbH, Mannheim, Germany). Using the bicinchoninic acid method (Price Rapid Gold BCA, Protein Assay Kit, Thermo Scientific, Waltham, MA, USA), the concentration of total protein was assessed. Samples reconstructed in Laemmli buffer were separated in the electrophoresis process and transferred into membranes. The membranes, according to manufacturer protocol, were blocked in EveryBlot Blocking Buffer (Bio-Rad Laboratories, Inc., Tokyo, Japan) and incubated overnight at 4 °C with antibodies as follows: anti-SIRT1 (1:1000), anti-SGLT2 (1:1000), anti-phosphorylated AMPK (pAMPK; 1:1000), and anti-AMPK (1:1000). To detect specific proteins, secondary antibodies (1:3000) were used. After incubation with proper secondary antibodies, the bands were detected with chemiluminescent substrate for Western blotting (Cyanagen, Bologna, Italy). The obtained specific protein bands were quantified densitometrically using a ChemiDoc visualization system (Image Laboratory Software Version 6.0.1; Bio-Rad, Warsaw, Poland).

### 2.5. Drugs

Dimethyl sulfoxide (DMSO) (Sigma-Aldrich, St. Louis, MO, USA); pentobarbitone sodium (Biowet, Puławy, Poland); and potassium chloride (KCl) (Chempur, Piekary Śląskie, Poland). Acetylcholine chloride and (−)-phenylephrine hydrochloride (Sigma, Munich, Germany) were dissolved in deionized water. A stock solution of U46619 (9,11-methanoepoxy PGH2) (Tocris, Bristol, UK) and EMPA (Ambeed, Arlington Heights, IL, USA) was prepared in ethanol or DMSO (0.1% *v*/*v*), respectively, and diluted with deionized water, which adjusted the final concentrations of ethanol and DMSO to less than 0.01% *v*/*v*.

EX527 (1 μM) and dorsomorphin were purchased from Ambeed, Arlington Heights, IL, USA, and stock solutions were prepared in DMSO. None of the solvents used affected basal parameters. The antibodies used in the Western blots were purchased from Abcam, Cambridge, UK; anti-SIRT1, anti-SGLT2, anti-pAMPK, anti-AMPK, and anti-rabbit or anti-mouse primary and anti-goat IgG horseradish peroxidase-conjugate secondary antibodies.

### 2.6. Statistical Analysis

Relaxation effects produced by EMPA and Ach were expressed as the percentage relaxation of the tone induced by Phe. Contractile responses to U46619 and Phe are presented as a percentage of the reference concentration-dependent vasoconstriction response to 60 mM of KCl after the equilibration period at the beginning of each experiment. The GraphPad Prism 10.4.1 software (San Diego, CA, USA) was used to plot the mean data as sigmoidal CRCs. Data were calculated for Gaussian distribution before statistical analysis. If the data were normally distributed, the one-way analysis of variance (ANOVA) with Dunnett’s multiple comparison test for multiple groups was carried out. The Student’s *t*-test for unpaired data was used as appropriate. All of the results are expressed as the mean ± SEM of n animals (n animals also corresponding to the number of independent experiments). The curves were then used to determine potency (pEC50, the negative logarithm of the concentration causing the half-maximum effect) and the maximum effect (Emax) values as the effects of the highest agonist concentration (determined graphically from the individual CRCs). Differences were considered significant at *p* < 0.05.

## 3. Results

### 3.1. Systolic Blood Pressure

SBP of the SHR measured by the tail cuff method was higher than the age-matched WKY (176.8 ± 5.2 mmHg; n = 10 vs. 103.0 ± 3.7 mmHg; n = 10, **** *p* < 0.0001; n = 10 respectively).

### 3.2. General

The vasorelaxant effects of EMPA in sMAs and acetylcholine (Ach) in sMAs and aortas were studied in vascular rings precontracted with phenylephrine (Phe; 10 μM, and 0.3 μM, respectively, a concentration approximately equivalent to its EC60). In all the experimental groups, WKY and SHR, the Phe-induced vasoconstriction (in mN) was comparable in sMAs (11.2 ± 2.3, n = 46; 12.1 ± 1.8, n = 46) and aortas (6.6 ± 0.5, n = 30; 6.5 ± 0.3, n = 30). The mean tensions (in mN) induced by 60 mM KCl were similar between the WKY and SHR groups in sMAs (9.9 ± 1.4, n = 80; 10.2 ± 1.1, n = 80) and aortas (11.4 ± 1.0, n = 20; 10.5 ± 0.6, n = 20). All of the inhibitors were present during the construction of CRCs and did not influence the preconstricted tone.

### 3.3. Influence of Hypertension on the Vasorelaxant Effect of EMPA in sMAs

**Figure 2:**
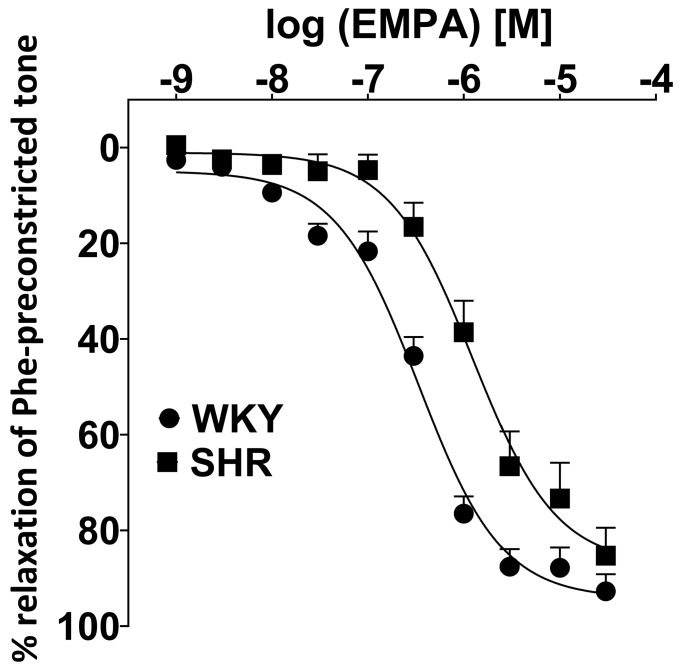
The vasorelaxant effects of empagliflozin (EMPA) in the endothelium-intact third-order of the superior mesenteric arteries (sMAs) isolated from Wistar Kyoto rats (WKY) and spontaneously hypertensive rats (SHR). Results are expressed as a percentage of relaxation of the isometric vasoconstriction induced by phenylephrine (Phe, 10 μM). Data are presented as the mean ± SEM of n = 6 for each curve.

EMPA (0.001–30 μM) produced a concentration-dependent relaxation of endothelium-intact sMAs preconstricted with Phe of normotensive and hypertensive rats. The vasorelaxant potency of EMPA was reduced in SHR compared to WKY (pEC50 = 5.9 ± 0.1, n = 6 vs. pEC50 = 6.5 ± 0.1; *p* < 0.01), since efficacy was similar in both groups (Emax = 85.2 ± 5.8, n = 6 vs. Emax = 92.7 ± 3.5, n = 6). CRCs of EMPA elicited rightward shifts about 4-fold in hypertensive rats (Figure 2).

### 3.4. Influence of Hypertension on EMPA-Mediated Changes in Ach-Induced Relaxation in sMAs and Aortas

**Figure 3:**
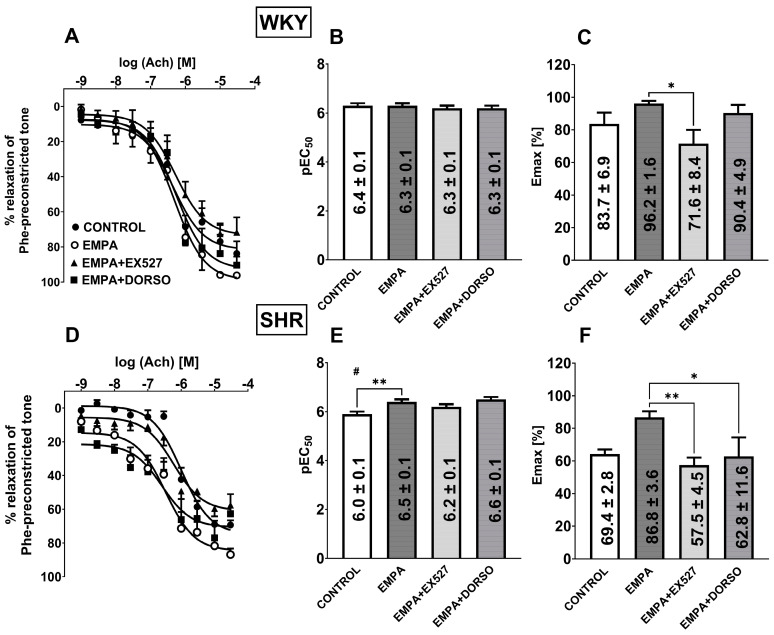
Influence of empagliflozin (EMPA; 10 µM) on the vasorelaxant effects of acetylcholine (Ach) in the presence of sirtuin 1 (SIRT1) inhibitor EX527 (1 µM) or AMP-activated protein kinase (AMPK) inhibitor dorsomorphin (DORSO; 10 µM) in the endothelium-intact third-order of the superior mesenteric arteries (sMAs) isolated from normotensive Wistar Kyoto rats (WKY, **A**–**C**) and spontaneously hypertensive rats (SHR, **D**–**F**). Vasodilatory responses are shown as a percentage of the reference response of the isometric concentration-dependent vasoconstriction induced by phenylephrine (Phe, 10 μM). For the statistical analysis, see the bar graph with values that present pEC50 (**B**,**E**) and maximal effect (Emax; **C**,**F**) obtained from each concentration-response curve. The results are presented as the mean ± SEM of n = 10 for each curve. * *p* < 0.05; ** *p* < 0.01 compared to the Ach + EMPA; # *p* < 0.05 compared to the WKY; as determined by one-way ANOVA followed by Dunnett’s multiple comparison test and Student’s *t*-test for unpaired data.

**Figure 4:**
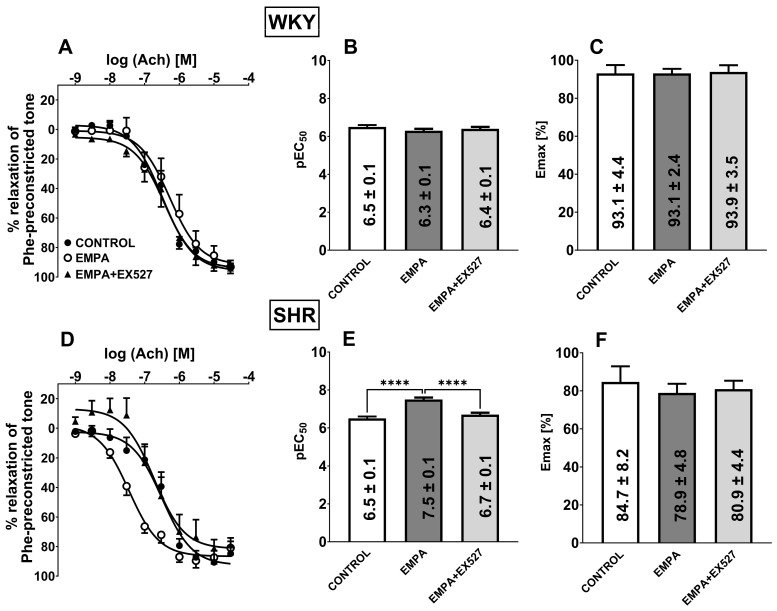
Influence of empagliflozin (EMPA; 10 µM) on the vasorelaxant effects of acetylcholine (Ach) in the presence of sirtuin 1 (SIRT1) inhibitor EX527 (1 µM) in the endothelium-intact aortas isolated from normotensive, Wistar Kyoto rats (WKY, **A**–**C**) and spontaneously hypertensive rats (SHR, **D**–**F**). Vasodilatory responses are shown as a percentage of the reference response of the isometric concentration-dependent vasoconstriction induced by phenylephrine (Phe, 0.3 μM). For the statistical analysis, see the bar graph with values that present pEC50 (**B**,**E**) and maximal effect (Emax; **C**,**F**) obtained from each concentration-response curve. The results are presented as the mean ± SEM of n = 10 for each curve. **** *p* < 0.0001 compared to the Ach + EMPA, as determined by one-way ANOVA followed by Dunnett’s multiple comparison test for unpaired data.

Ach (0.001–30 µM) produced a concentration-dependent relaxation of endothelium-intact sMAs (Figure 3A,D) and aortas (Figure 4A,D) preconstricted with Phe of normotensive and hypertensive rats. Ach exhibited decreased affinity only in sMAs isolated from hypertensive rats vs. normotensive animals (Figure 3B,E). Efficacy of aortas was similar in both groups; however, in sMAs, it showed a downward trend in SHR (*p* = 0.0708) (Figure 3C,F). Incubation with EMPA (10 μM) enhanced the potency of Ach-mediated relaxation in sMAs (Figure 3D,E) and aortas (Figure 4D,E) in the SHR group without affecting the maximum relaxation response (Figure 3D,F and Figure 4D,F). Pre-treatment with EMPA shifted the CRC for Ach to the left in sMAs and aortas by a factor of 3 and 10, respectively (for the pEC50, Emax values, and statistical analyses, see Figure 3B,C,E,F and Figure 4B,C,E,F).

### 3.5. The Role of SIRT1 on EMPA-Mediated Changes in Ach-Induced Relaxation in sMAs and Aortas

The combination of EMPA (10 μM) and selective SIRT1 inhibitor EX527 (1 µM) reduced Emax of Ach-induced relaxation in sMAs isolated from WKY and SHR groups by about 25% and 35% (Figure 3C,F), but not in aortas (Figure 4C,F) in comparison with the EMPA group. Pre-treatment with EMPA and EX527 attenuated potency in aortas isolated from SHR (Figure 4E) in comparison with the EMPA group and shifted the CRC for Ach to the right by a factor of 6 (Figure 4D).

### 3.6. The Role of AMPK on EMPA-Mediated Changes in Ach-Induced Relaxation in sMAs

Combined administration of EMPA (10 μM) and the AMPK inhibitor DORSO (10 µM) reduced the efficacy of about 30% of Ach-induced relaxation in sMAs isolated from SHR in comparison with the EMPA group (Figure 3F).

### 3.7. Influence of Hypertension on EMPA-Mediated Changes in Phe-Induced Vasoconstriction in sMAs and Aortas

**Figure 5:**
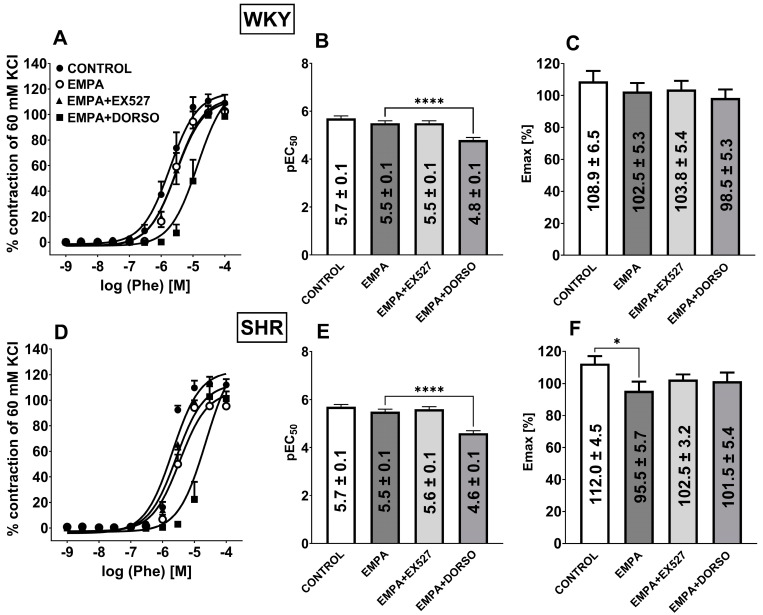
Influence of empagliflozin (EMPA; 10 µM) on the vasocontractile effects of phenylephrine (Phe) in the presence of sirtuin 1 (SIRT1) inhibitor EX527 (1 µM) or AMP-activated protein kinase (AMPK) inhibitor dorsomorphin (DORSO; 10 µM) in the endothelium-intact third-order of the superior mesenteric arteries (sMAs) isolated from Wistar Kyoto rats (WKY, **A**–**C**) and spontaneously hypertensive rats (SHR, **D**–**F**). Contractile responses are shown as a percentage of the reference response of the isometric concentration-dependent vasoconstriction induced by 60 mM KCl. For the statistical analysis, see the bar graph with values that present pEC50 (**B**,**E**) and maximal effect (Emax; **C**,**E**) obtained from each concentration-response curve. The results are presented as the mean ± SEM of n = 10 for each curve * *p* < 0.05; **** *p* < 0.0001 compared to the Phe + EMPA, as determined by one-way ANOVA followed by Dunnett’s multiple comparison test for unpaired data.

**Figure 6:**
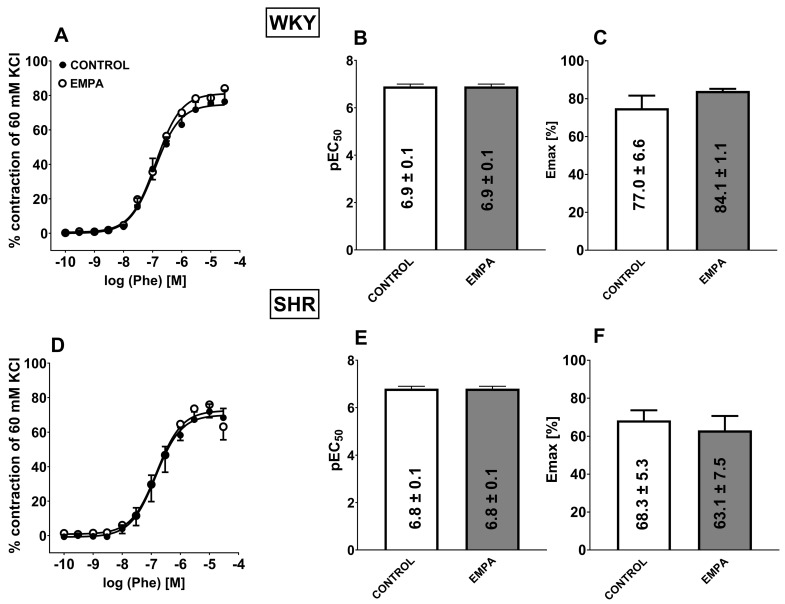
Influence of empagliflozin (EMPA; 10 µM) on the vasocontractile effects of phenylephrine (Phe) in the endothelium-intact aortas isolated from normotensive Wistar Kyoto rats (WKY, **A**–**C**) and spontaneously hypertensive rats (SHR, **D**–**F**). Contractile responses are shown as a percentage of the reference response of the isometric concentration-dependent vasoconstriction induced by 60 mM KCl. For the statistical analysis, see the bar graph with values that present pEC50 (**B**,**E**) and maximal effect (Emax; **C**,**E**) obtained from each concentration-response curve. The results are presented as the mean ± SEM of n = 10 for each curve.

Phe (0.001–30 µM) induced concentration-dependent vasoconstriction of sMAs (Figure 5A,D) and aortas (Figure 6A,D) isolated from WKY and SHR. Administration of 10 µM EMPA reduced only the Emax by about 15% but not the potency in sMAs of hypertensive rats (Figure 5D). No changes in response to Phe were observed in the aortas isolated from WKY and SHR (Figure 6A–F).

Combined administration of EMPA (10 μM) and the AMPK inhibitor DORSO (10 µM) decreased the affinity of Phe-induced concentration-dependent vasoconstriction in sMAs in WKY and SHR groups compared to the EMPA group and shifted the CRC for Phe to the right by a factor of 5 and 8, respectively (Figure 5A,B,D,E).

### 3.8. Influence of Hypertension on EMPA-Mediated Changes in Analog Thromboxane U46619-Induced Vasoconstriction in sMAs

**Figure 7:**
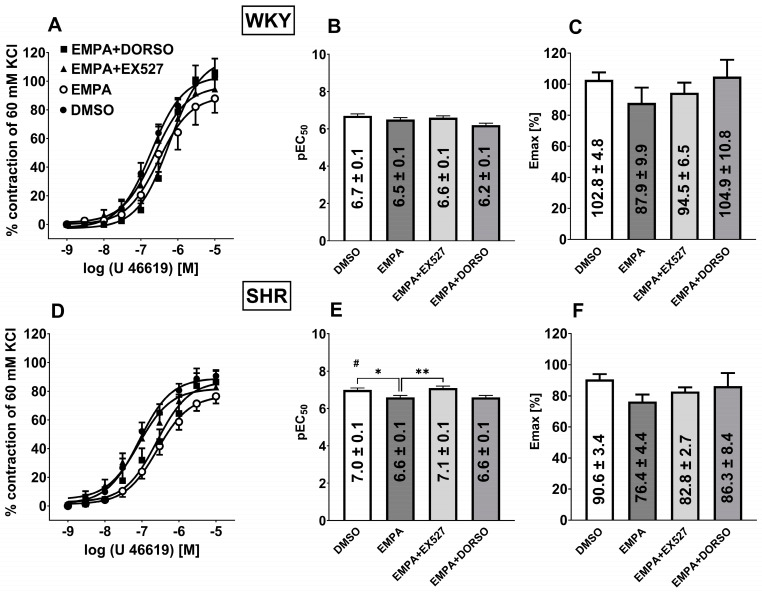
Influence of empagliflozin (EMPA; 10 µM) on the vasocontractile effects of thromboxane analogue U46619 in the presence of sirtuin 1 (SIRT1) inhibitor EX527 (1 µM) or AMP-activated protein kinase (AMPK) inhibitor dorsomorphin (DORSO; 10 µM) in the endothelium-intact third-order of the superior mesenteric arteries (sMAs) isolated from Wistar Kyoto rats (WKY, **A**–**C**) and spontaneously hypertensive rats (SHR, **D**–**F**). Contractile responses are shown as a percentage of the reference response of the isometric concentration-dependent vasoconstriction induced by 60 mM KCl. For the statistical analysis, see the bar graph with values that present pEC50 (**B**,**E**) and maximal effect (Emax; **C**,**E**) obtained from each concentration-response curve. The results are presented as the mean ± SEM of n = 10 for each curve. * *p* < 0.05; ** *p* < 0.01 compared to the U46619 + EMPA; # *p* < 0.05 compared to the WKY; as determined by one-way ANOVA followed by Dunnett’s multiple comparison test and Student’s *t*-test for unpaired data.

The thromboxane analog U46619 (0.001–10 µM) induced concentration-dependent vasoconstriction of the sMAs (Figure 7A,D) isolated from WKY and SHR.

The vasoconstrictor potency was higher in sMAs isolated from hypertensive rats compared to the normotensive group (Figure 7B,E), whereas the efficacy showed a trend toward being reduced in SHR (*p* = 0.0527) (Figure 7C,F). The presence of EMPA (10 μM) in the SHR group reduced the affinity of U46619-induced sMAs concentration-dependent vasoconstriction compared to the control group (Figure 7E).

Combined administration of EMPA and EX527 (1 µM) increased the affinity of U46619-induced concentration-dependent vasoconstriction in sMAs of SHR compared to the EMPA group and shifted the CRC for U46619 to the left by a factor of 3 (Figure 7D,E).

### 3.9. Influence of Hypertension on the Expression of SGLT2, SIRT1, AMPK, and pAMPK in Isolated sMAs and Aortas

**Figure 8:**
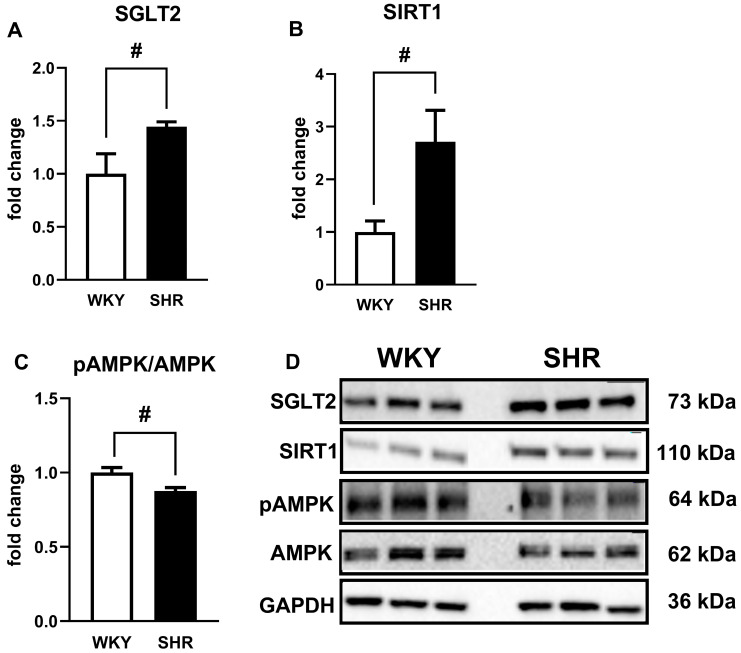
The relative expression of a sodium-glucose co-transporter-2 (SGLT2) (**A**), sirtuin 1 (SIRT1) (**B**) and the phosphorylated form of adenosine monophosphate-activated protein kinase/adenosine monophosphate-activated protein kinase (pAMPK/AMPK) (**C**) and their representative bands (**D**) evaluated by Western blot in the third-order of the superior mesenteric arteries (sMAs) of Wistar Kyoto rats (WKY) and spontaneously hypertensive rats (SHR). Full-length Western blot images are shown in Figure S1. Data are presented as mean ± SEM (n = 5–6 per group); # *p* < 0.05 compared to the control, where expression level was set as 1. Glyceraldehyde-3-phosphate dehydrogenase (GAPDH) served as a loading control.

**Figure 9:**
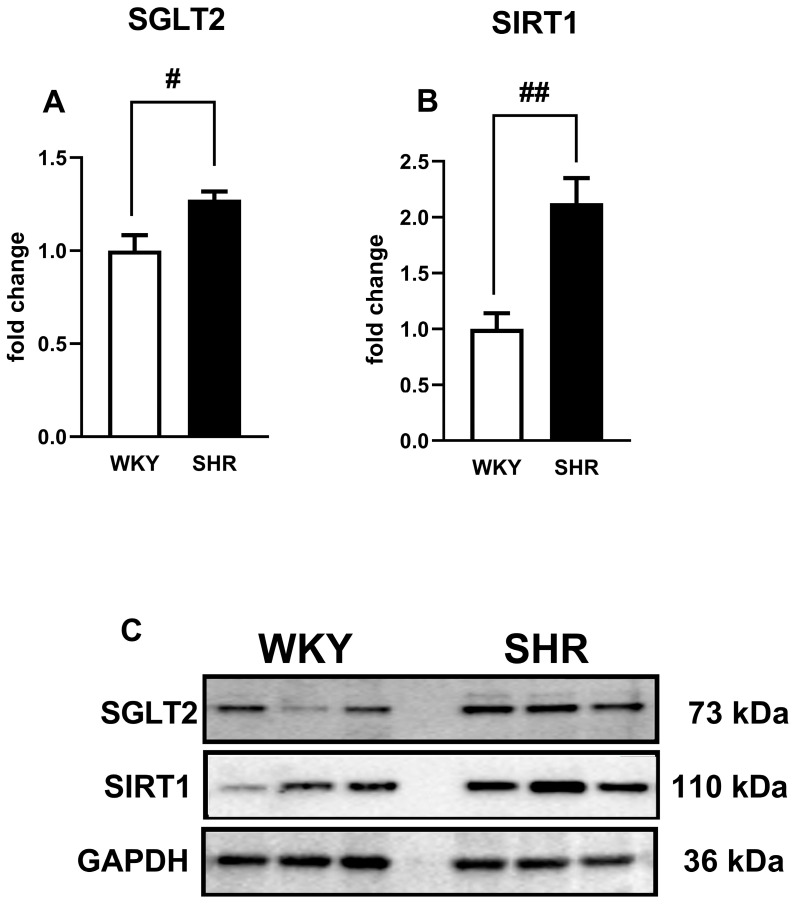
The relative expression of a sodium-glucose co-transporter-2 (SGLT2) (**A**), sirtuin 1 (SIRT1) (**B**), and their representative bands (**C**) evaluated by Western blot in aortas of Wistar Kyoto rats (WKY) and spontaneously hypertensive rats (SHR). Full-length Western blot images are shown in Figure S2. Data are presented as mean ± SEM (n = 5–6 per group); # *p* < 0.05; ## *p* < 0.01 compared to the control, where the expression level was set as 1. Glyceraldehyde-3-phosphate dehydrogenase (GAPDH) served as a loading control.

The expression of SGLT2, SIRT1, AMPK, and pAMPK in isolated sMAs and aortas was analyzed by the Western blot method. It showed a single immunoreactive band of the molecular size expected for SGLT2 (73 kDa), SIRT1 (110 kDa), AMPK (62 kDa), and pAMPK (64 kDa) in both types of vessels (Figure 8D and Figure 9C). In hypertensive rats, the SGLT2 and SIRT1 expression was significantly increased in sMAs by about 45% and 170% (Figure 8A,B) and in aortas by about 30% and 120%, respectively (Figure 9A,B). The expression of pAMPK/AMPK was decreased by nearly 15% in sMAs from SHR compared to WKY (Figure 8C).

## 4. Discussion

We examined whether EMPA plays a vasoprotective and modulatory role under hypertensive conditions. We also investigated whether SIRT1 and AMPK are involved in EMPA-mediated regulation of vasomotor tone in this pathological state. For this purpose, we used the most common animal model of primary hypertension, SHR, which reflects human essential hypertension and is connected with impaired endothelial function [29], and normotensive controls, WKY.

We examined conduit (aortas) and resistance (sMAs) vessels because hypertension and SGLT2 inhibitors induce vascular changes depending on the vessel size [33]. Nevertheless, most experiments focused on resistance mesenteric arteries since these arteries play a crucial role in blood pressure control [34]. Myography and organ baths are most commonly used to assess vascular function directly, but wire myography offers better access to the endothelium [30].

The blood pressure lowering effect of SGLT2 inhibitors appears to be a dose-independent class effect; however, the mechanism underlying this effect is not fully known. In this study, we observed an increased expression of SGLT2 both in sMAs and aortas from SHR. SGLT2 expression was also demonstrated in third- and fourth-order branches of the superior mesenteric arteries [15], which is in line with our research. Moreover, it was shown a tendency to increase the expression of SGLT2 in aortas in the model of abdominal aortic aneurysm, where angiotensin II infusion in the ApoE-/- mouse model elicits aortic media destruction and inflammatory cell infiltration [35]. In our study, we observed attenuated vasodilation to Ach and an increased efficiency of U46619 in sMAs of hypertensive rats compared to WKY, which is probably associated with endothelial dysfunction. SGLT2 expression increases under hypertensive conditions, possibly as a compensatory mechanism for elevated vascular tone, especially at an early stage of the disease [36].

### 4.1. EMPA Relaxed Endothelium-Intact sMAs

In this study, we demonstrated for the first time that EMPA mediated the concentration-dependent full relaxation in the endothelium-intact sMAs preconstricted with Phe in SHR. The pEC50 value for its vasodilatory response was in the mid-micromolar range (~6.0). The relaxation is reduced when compared with normotensive control (pEC50 ~6.5). It could also be extrapolated to the in vivo therapeutic concentrations of EMPA in serum after oral administration of a common dose of 10 mg in humans, which is approximately 0.3 μM. In addition, EMPA at 800 mg/day reached a plasma concentration of 8 μM [37]. Ex vivo studies often employ higher drug concentrations to identify cellular processes [10]. In these findings, we observed a ~30-fold higher potency compared to the results of previous investigations conducted in the third- and fourth-order of the superior mesenteric arteries of Sprague Dawley rats [14], second-order mesenteric arteries from Wistar rats (pEC_50_ ~5.0) [10], and conduit arteries, such as coronary arteries isolated from Sprague Dawley rats (pEC_50_ ~5.0) [16]. Other studies have shown even weaker potency of the direct vasorelaxant effect of EMPA in aortas of rabbits (pEC_50_ ~3.8) [17]. Existing reports attempt to explain this phenomenon, with conflicting findings. Some studies suggest that EMPA inhibits kinase G activity [14,17,38] and/or activates Kv1.5 and Kv7 channels [14,15], while others have not confirmed these effects [19]. Additionally, EMPA has been linked to the inhibition of sodium/hydrogen exchangers and decreased aortic levels of angiotensin II and endothelin-1 in the rheumatoid arthritis/diabetes mellitus co-morbidity rats [39]. Studies have also demonstrated that EMPA induced vasorelaxation independently of the endothelium [10,14,15,17,38]. It is important to note, however, that the efficacy and mechanisms of action of SGLT2 inhibitors vary across different animal strains and vascular beds, including both resistance and conduit arteries, due to their structural and functional properties.

### 4.2. EMPA Improved Vascular Function in Hypertension

To define EMPA’s effects as a modulator of hypertensive vascular tone, we investigated its impact on vascular reactivity in sMAs and aortas isolated from SHR. For the first time, our data shows that acute administration of EMPA (10 µM) before performed CRCs significantly enhanced vasodilation responses to Ach in sMAs as well as aortas preconstricted with Phe under hypertensive conditions only. Our results are in line with research where the same concentration of EMPA improved relaxation to Ach of human internal mammary arteries stimulated with glucose or angiotensin II [40]. In normotension, canagliflozin (10 µM and 50 µM), another inhibitor of SGLT2, also enhanced vasorelaxant response to Ach of thoracic aortas from Sprague Dawley rats and after vascular ischemia/reperfusion injury in Wistar rats and mesenteric arteries in Dahl sensitive rats ([41,42,43], respectively). Therefore, flozins, including EMPA, improve endothelium-dependent relaxation irrespective of vessel function or diameter.

Results from chronic SGLT2 inhibitor administration are consistent with those found in acute experiments. By targeting the cardiovascular system, specifically endothelial function, EMPA and other inhibitors of SGLT2 showed significant benefits. In diabetic conditions, EMPA (20 mg/kg/day) improved endothelial dysfunction, as determined by Ach-induced vasodilation [44]. In addition, treatment with canagliflozin (20 and 30 mg/kg/day) or dapagliflozin (1 mg/kg/day) ameliorated endothelial dysfunction, as determined by Ach-induced vasodilation in mesenteric arteries or thoracic aortas [19,43].

To our knowledge, this is the first observation that EMPA attenuated the enhanced contractile effects of Phe and U46619 in SHR only. Accordingly, canagliflozin (10 µM) reduced the promoted vasoconstriction induced by Phe or U46619 in Dahl-sensitive rats on a long-term high salt diet. Further, this inhibitory effect of canagliflozin on vasoconstriction pathways is independent of NO but dependent on transient receptor potential channel 3 (TRPC3). Vasoconstriction was enhanced by a high salt diet only in Dahl sensitive rats, suggesting that enhanced constriction plays a critical role in salt-sensitive hypertension. According to the authors, canagliflozin’s antihypertensive effect is mainly mediated by reduced vasoconstriction with a key role for TRPC3 but not vasodilation despite improved endothelial function [43]. However, the dilatation mechanism of these phenomenon requires further investigation.

### 4.3. SIRT1 Is Involved in EMPA-Mediated Vascular Protection in Hypertension

For the first time, we showed that SIRT1 expression is increased in isolated sMAs and aortas of SHR compared to WKY rats. It is in line with functional studies, in which combined administration of SIRT1 inhibitor (EX527) abolished the beneficial effects of EMPA, i.e., (1) Ach-induced vasodilation in sMAs in both groups of rats and thoracic aortas in SHR and (2) reduced contractile response to U46619 in SHR but not WKY. Similarly, pharmacological (EX527 or spirinol) or adenoviral (siRNA) inhibition of SIRT1 induced endothelial dysfunction, as shown by the reduced relaxation to the endothelium-dependent vasodilator, Ach [20,45,46]. Moreover, Ach did not induce vasodilation in the presence of the NOS inhibitor NG-nitro-L-arginine methyl ester (L-NAME) even with SIRT1 overexpression. This suggests that SIRT1 does not activate NOS-independent mechanisms for endothelium-dependent relaxation. Therefore, endothelial SIRT1 promotes endothelium-dependent vasodilation and enhances the vascular bioavailability of NO through a NOS-dependent mechanism in cultured endothelial cells [20,47]. Therefore, changes in the function of SIRT1 significantly affect the vascular function associated with hypertension [48]. Emerging evidence suggests that the SIRT1 pathway might play a role in the protective effects of EMPA on vasculature [16].

### 4.4. AMPK Is Involved in EMPA-Mediated Vascular Protection in Hypertension

In our data, we observed decreased protein expression of pAMPK/AMPK in sMAs isolated from SHR. Co-incubation of EMPA with DORSO abolished the advantageous effect of EMPA on the vasodilatory response to Ach in sMAs isolated from SHR and reduced the vasoconstriction induced by Phe. Numerous studies have shown that the development of hypertension is related to dysfunction of the AMPK signaling pathway [49,50], which correlated with our Western blot results. Despite AMPK activity being found to be also decreased in the aortas of SHR, the AMPK activator (AICAR) or metformin could directly lower blood pressure and generate both endothelium-dependent and -independent relaxation in arteries from WKY and an enhanced relaxant effect in arteries from SHR. These results suggested that AMPK may be a useful target for improving the vascular dysfunction that exists in cardiovascular disease states such as hypertension or other disease states exhibiting vasomotor dysfunction due to impaired NO-dependent relaxation and/or enhanced cyclooxygenase-mediated concentration-dependent constriction [51,52]. It could be assumed that blocking AMPK will increase vasoconstriction, but in our hands, we did not observe the action of EMPA via AMPK in U46619-induced concentration-dependent constriction and even significant attenuation, as is the case with vasoconstriction induced by Phe. One explanation of this phenomenon is that DORSO inhibits several other kinases much more potently than AMPK and is therefore highly non-specific [53]. The present results do not permit further speculation of the mechanism by which DORSO inhibits the transduction of the contractile process, especially to α1-adrenergic agonists. Further research is required, especially with more specific inhibitors available on the market.

### 4.5. SIRT1/AMPK Crosstalk in Vascular Protection in Hypertension

SGLT2 inhibitors upregulate SIRT1 and AMPK pathways through a crosslink of mutual activation and are described in metabolic diseases [23,54,55]. In addition, EMPA via SIRT1/AMPK connection may contribute to cellular protection in various tissues, including the heart, brain, liver and others [56,57,58]. However, this phenomenon is poorly understood in the vasculature. Activation of AMPK and SIRT1 has been shown to ameliorate hypertensive vascular remodeling [59] and improve NO-dependent vasodilation in the aorta, leading to reduced blood pressure [50]. Moreover, Zhai et al. [55] indicated that activation of SIRT1 and AMPK by EMPA represents a potential therapeutic strategy for preeclampsia in mice. Given the pivotal role of the SIRT1/AMPK axis in vascular health, we hypothesize that modulation of these pathways by EMPA may offer protective effects against hypertensive vascular dysfunction.

Recent studies suggest that microRNAs (miRNAs) may play a crucial role in the regulation of the SIRT1/AMPK axis, further influencing vascular homeostasis [60] Overexpression of miR-34a has been shown to downregulate SIRT1, thereby accelerating aging and oxidative stress-induced damage in retinal vessels of diabetic rats [61,62] and inhibiting AMPK signaling, leading to disturbances in cellular energy balance and stress adaptation mechanisms [63]. These findings underscore the intricate interplay between miRNAs, SIRT1, and AMPK in vascular function and disease progression. miRNAs can regulate SIRT1 activity, which in turn modulates AMPK-driven metabolic pathways [60].

Chronic administration of EMPA exhibits a hypotensive effect in SHR [64] and humans [65] and may further enhance SIRT/AMPK expression. This could lead to sustained cardiovascular benefits by amplifying protective mechanisms associated with SIRT/AMPK upregulation or by acting through upstream regulators and/or involving miRNA,which requires thorough investigation.

## 5. Limitations

Our study has several limitations. First of all, we have done our best to use the selective and common ligands [26,31] however, DORSO has also been suggested to inhibit other kinases. At concentration 10 µM, DORSO inhibits AMPK by 90%. However, Vogt et al. [53] showed that DORSO at the same concentration additionally inhibited the activity of 64 of the other 119 kinases tested by more than 50%. Secondly, we acknowledge that performing additional control experiments with EX527 and DORSO (without EMPA) on Phe- and TXA2-induced contractions would provide a clearer distinction between the effects of EMPA and those of the inhibitors alone. This could help determine whether there are any additive or antagonistic effects in relation to EMPA’s actions. Thirdly, the effects of EMPA can vary between ages and sexes due to differences in hormonal regulation, cardiovascular physiology, and the expression of SGLT2 in tissues. The ability to optimize the therapeutic potential of SGLT2 inhibitors in both men and women requires an understanding of these age- and gender-based differences [63,66]. It would be interesting to evaluate whether standard guideline-based therapy is more effective than EMPA-based endothelial (vascular) function-focused therapy. Furthermore, it would be advisable to investigate if observed vascular effects are not only an individual effect of EMPA but rather a class effect of the flozins; one experimental series with another flozin would be helpful. To properly assess the vasoprotective effects of EMPA on blood vessels, chronic studies should be conducted, along with the evaluation of flow-related genes such as eNOS, nuclear factor erythroid 2-related factor 2, vascular endothelial growth factor, and proteins that confirm endothelial integrity, such as intercellular adhesion molecule-1, vascular endothelial cadherin, and vascular cell adhesion molecule-1, as well as pro B-type natriuretic peptide or atrial natriuretic peptide, which can reflect cardiac stress and vascular health.

## 6. Conclusions

**Figure 10:**
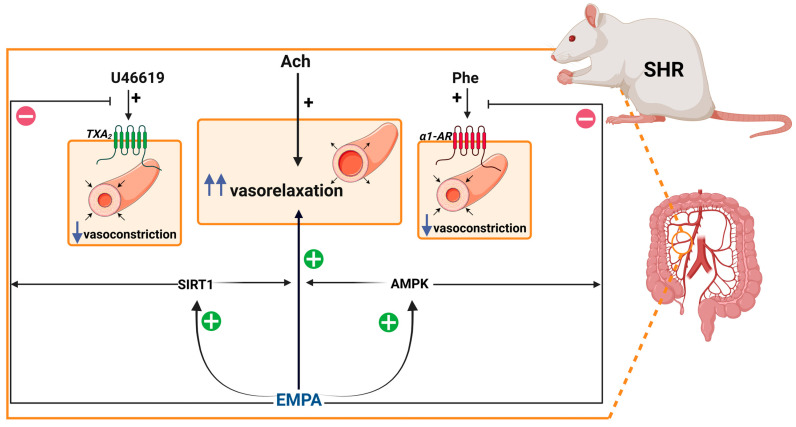
Proposed mechanism of vasoprotective actions of empagliflozin (EMPA) in the third-order of the superior mesenteric arteries (sMAs) in spontaneously hypertensive rats (SHR). EMPA enhances acetylcholine (Ach)-induced relaxation acting via sirtuin 1 (SIRT1) and kinase AMP-activated protein (AMPK). The phenylephrine (Phe)-induced concentration-dependent vasoconstriction is inhibited by the influence of AMPK and U46619-mediated concentration-dependent vasoconstriction by SIRT1. Created with BioRender.com.

In conclusion, EMPA, a member of the SGLT2 inhibitor class often called “the statins of the 21st century” [67], offers new insights into the mechanisms underlying vascular dysfunction in hypertension, as shown by the results of this study. Specifically, here we provide for the first time that EMPA (1) has a direct concentration-dependent vasodilatory effect on the sMAs and might play a vasoprotective role in SHR and (2) alleviates hypertension-related vascular changes through AMPK/SIRT1 signaling. Therefore, EMPA enhanced endothelium-dependent vasodilation to Ach in aortas and sMAs via SIRT1 and AMPK. Moreover, in resistance but not in conduit arteries, it also attenuated enhanced vasoconstriction to α1-adrenergic through AMPK and/or thromboxane A2 agonist by SIRT1 (Figure 10). This suggests that the phenomenon may be agonist and vessel type dependent. Moreover, given the emerging evidence linking microRNAs to key regulators of vascular homeostasis, such as SIRT1 and AMPK, further investigation into their and other upstream molecules involvement in mediating the cardiovascular benefits of flozins is warranted. Unraveling these pathways could provide novel therapeutic insights and pave the way for more targeted approaches in vasoprotection in vascular injury-related diseases, including hypertension, diabetes and others. A further translational study is required to optimize EMPA’s and other SGLT2 inhibitors’ clinical application in alleviating hypertension-related vascular complications.

## 7. Perspectives

Flozins are increasingly prescribed due to their significant cardiometabolic benefits in people with diabetes, heart failure, and chronic kidney disease. Their pleiotropic effects are particularly important, as they may help reduce the total number of medications a patient needs to take. This, in turn, can lower the risk of correlated drug-related adverse effects in individuals with multimorbidity [68]. However, further research is needed to clarify whether chronic EMPA targets SIRT1/AMPK or operates through upstream regulators and/or involving miRNA. A deeper insight into its role in arterial vasodilation could expand the future application of SGLT2 inhibitors and broaden their therapeutic potential in vascular health.

## Data Availability

The data presented in this study are available on request from the corresponding authors.

## Supplementary Materials

The following supporting information can be downloaded at: https://www.mdpi.com/article/10.3390/cells14070507/s1, Figure S1: Uncropped Western blots shown in Figure 7. Abbreviations: AMPK—AMP-activated protein kinase; pAMPK—phosphorylated form AMP-activated protein kinase; SGLT2—a sodium-glucose cotransporter-2; SHR—spontaneously hypertensive rats; SIRT1-sirtuin 1; sMAs—the third-order of the superior mesenteric artery; WKY—Wistar-Kyoto rats. The black arrows indicate specific molecular weights obtained using the special Western blot standard which help identify and characterize the molecules separated in a gel. The red arrows indicate bands detected with the primary antibodies. The blue rectangles show bands that have been selected to be shown in original Figure 7 in manuscript. Figure S2: Uncropped Western blots shown in Figure 8. Abbreviations: SGLT2—a sodium-glucose cotransporter-2; SHR—spontaneously hypertensive rats; SIRT1-sirtuin 1; sMAs—the third-order of the superior mesenteric artery; WKY—Wistar-Kyoto rats. The black arrows indicate specific molecular weights obtained using the special Western blot standard which help identify and characterize the molecules separated in a gel. The red arrows indicate bands detected with the primary antibodies. The blue rectangles show bands that have been selected to be shown in original Figure 8 in manuscript.

## Abbreviations

Ach	acetylcholine
AMPK	AMP-activated protein kinase
CRCs	cumulative concentration-response curves
DORSO	dorsomorphin
EDH	endothelial-derived hyperpolarization
EMPA	empagliflozin
eNOS	endothelial nitric oxide synthase
NIBP	Non-Invasive Blood Pressure
NO	nitric oxide
pAMPK	phosphorylated form AMP-activated protein kinase
Phe	phenylephrine
SBP	systolic blood pressure
SGLT2	a sodium-glucose co-transporter 2
SHR	spontaneously hypertensive rats
SIRT1	sirtuin 1
sMAs	the third-order of the superior mesenteric artery
WKY	Wistar Kyoto rats
