# Supplementary material for: Empagliflozin Plays Vasoprotective Role in Spontaneously Hypertensive Rats via Activation of the SIRT1/AMPK Pathway
Source: Cells. 2025 Mar 29;14(7):507. doi: 10.3390/cells14070507 (PMC11987869; doi:10.3390/cells14070507)
Supplement: Supplementary file 1 [file cells-14-00507-s001.zip › cells-3517967-supplementary.pdf]

<u>SUPPLEMENTARY MATERIAL</u>

Empagliflozin Plays Vasoprotective Role in Spontaneously Hypertensive Rats via Activation of the SIRT1/AMPK Pathway

Monika Kloza, Anna Krzyżewska, Hanna Kozłowska, Sandra Budziak and Marta Baranowska-Kuczko

Corresponding authors: monika.kloza@umb.edu.pl; marta.baranowska@umb.edu.pl

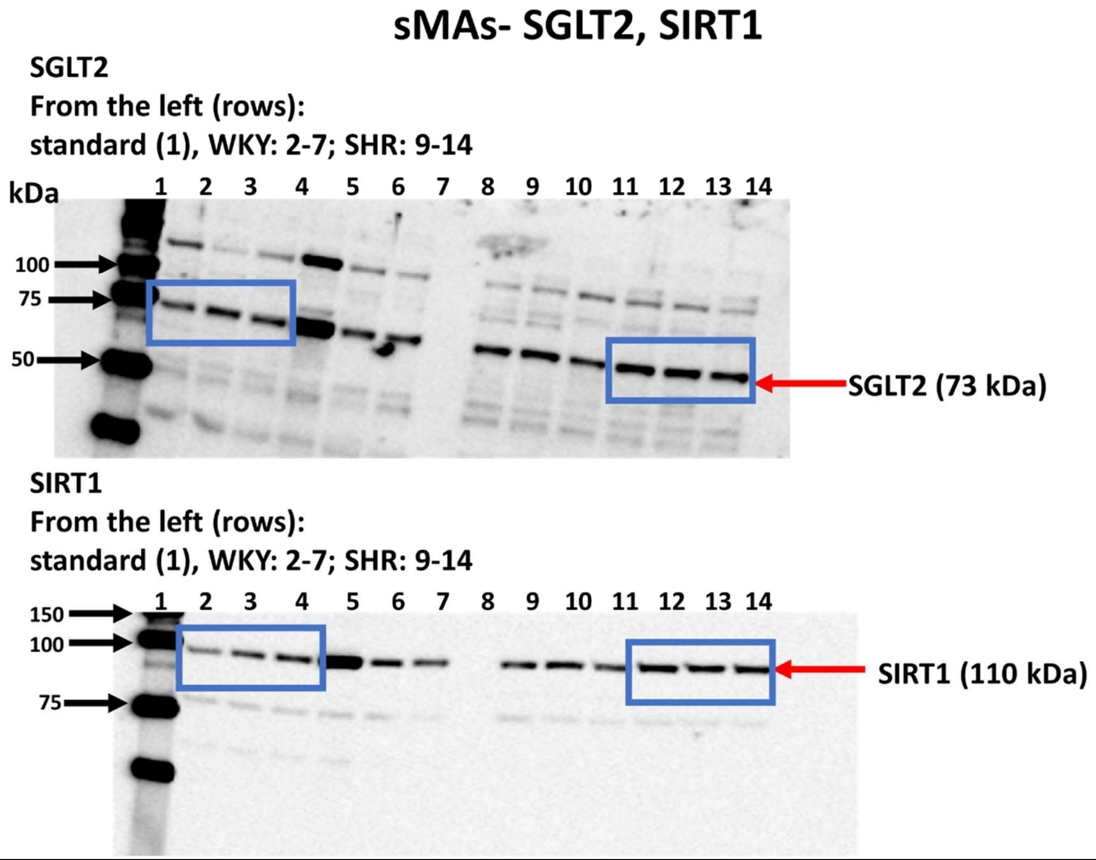

sMAs- pAMPK, AMPK

pAMPK
From the left (rows):
standard (1), WKY: 2-7; SHR: 9-14

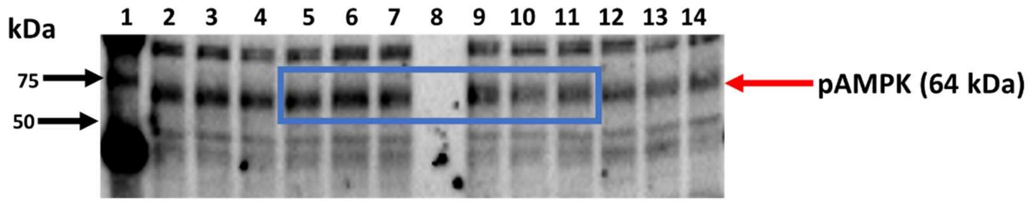

AMPK
From the left (rows):
standard (1), WKY: 2-7; SHR: 9-14

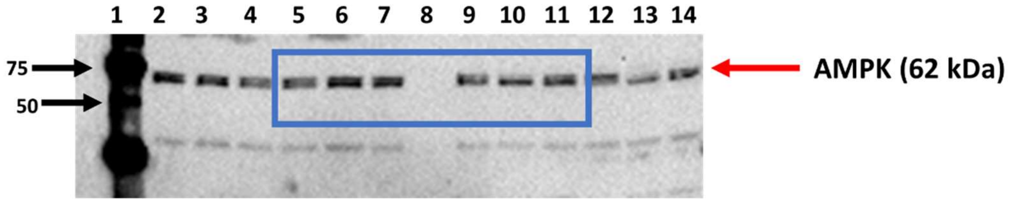

sMAs - GAPDH

The levels of the proteins detected were normalized to GAPDH.

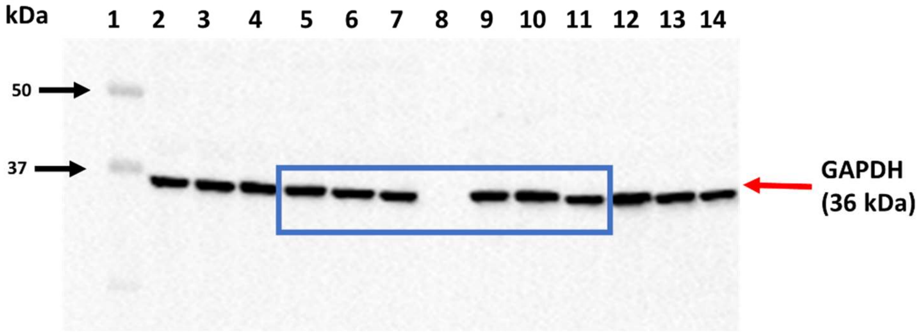

Figure S1. Uncropped Western blots shown in Fig.7. Abbreviations: AMPK - AMP-activated protein kinase; pAMPK - phosphorylated form AMP-activated protein kinase; SGLT2 - a sodium-glucose cotransporter-2; SHR - spontaneously hypertensive rats; SIRT1- sirtuin 1; sMAs - the third-order of the superior mesenteric artery; WKY - Wistar-Kyoto rats

The black arrows indicate specific molecular weights obtained using the special western blot standard which help identify and characterize the molecules separated in a gel.

The red arrows indicate bands detected with the primary antibodies.

The blue rectangles show bands that have been selected to be shown in original figure7 in manuscript.

Aorta- SGLT2, SIRT1

SGLT2
From the left (rows):
standard (1), WKY: 2-7; SHR: 9-14

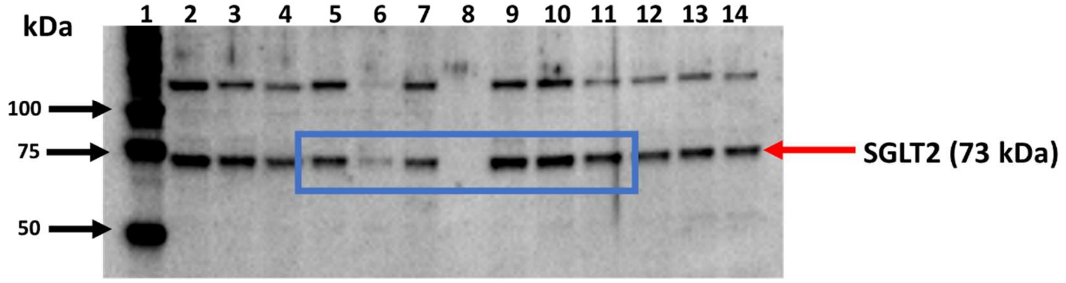

SIRT1
From the left (rows):
standard (1), WKY: 2-7; SHR: 9-14

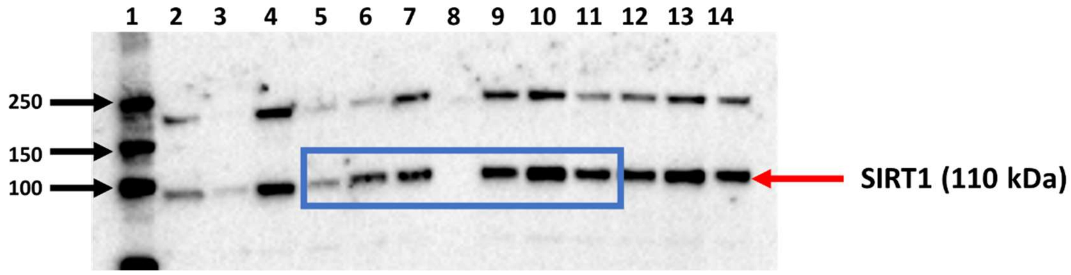

Aorta- GAPDH

The levels of the proteins detected were normalized to GAPDH.

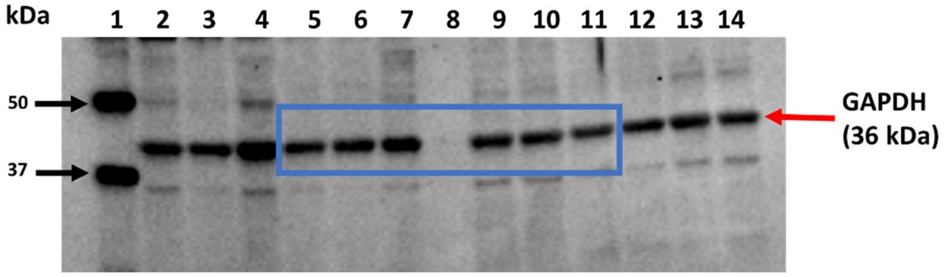

Figure S2. Uncropped Western blots shown in Fig.8. Abbreviations: SGLT2 - a sodium-glucose cotransporter-2; SHR - spontaneously hypertensive rats; SIRT1- sirtuin 1; sMAs - the third-order of the superior mesenteric artery; WKY - Wistar-Kyoto rats

The black arrows indicate specific molecular weights obtained using the special western blot standard which help identify and characterize the molecules separated in a gel.

The red arrows indicate bands detected with the primary antibodies.

The blue rectangles show bands that have been selected to be shown in original figure8 in manuscript.
